# Esophageal Variceal Grading and Platelet Count in Patients With Liver Cirrhosis

**DOI:** 10.7759/cureus.90816

**Published:** 2025-08-23

**Authors:** Muhammad Farooq, Usman Sadiq, Qaiser Wadud, Abbas Ali, Dania Khawar Rahman, Muhammad Imran, Mahnoor Safeer, Syeda Hafsa Habib, Tayaba Syed Habib

**Affiliations:** 1 Department of General Medicine, Nishtar Medical University, Multan, PAK; 2 Department of General Medicine, Tertiary Care Hospital Nishtar-II, Multan, PAK; 3 Department of General and Internal Medicine, Khyber Teaching Hospital, Peshawar, PAK; 4 Department of Internal Medicine, Lady Reading Hospital, Peshawar, PAK; 5 Department of Gastroenterology, Pakistan Institute of Medical Sciences (PIMS), Islamabad, PAK; 6 Department of Internal Medicine, Mohi-ud-Din Islamic Medical College and Mohi-ud-Din Teaching Hospital, Mirpur, PAK; 7 Medical School, Khyber Medical University (KMU) Institute of Medical Sciences (KIMS), Kohat, PAK; 8 Medical School, Swat Medical College (STMC), Swat, PAK

**Keywords:** endoscopy, esophageal varices, liver cirrhosis, pakistan, platelet count, risk assessment, thrombocytopenia

## Abstract

Background: Esophageal varices represent a critical consequence of liver cirrhosis, with the risk of hemorrhage closely associated with the grade of the varices. Upper gastrointestinal endoscopy (UGIE) is the definitive method for detection; nonetheless, it is invasive and resource-demanding. Recognizing straightforward, non-invasive indicators like platelet count may assist in risk stratification and prioritize endoscopic screening in patients with cirrhosis.

Objective: This study aimed to ascertain the correlation between esophageal variceal grading and platelet count in individuals with liver cirrhosis and to assess the clinical relevance of thrombocytopenia as a predictor of high-grade varices.

Materials and methods: This observational, descriptive study was performed from August 2022 to August 2023. One hundred twenty cirrhotic individuals had a UGIE assessment for esophageal varices. Demographic, clinical, and laboratory data, including platelet counts, were documented. Patients were classified according to platelet count (>150, 100-150, and <100×10⁹/L) and variceal grades (I-III). The correlation between platelet count and variceal grade was evaluated utilizing chi-squared tests, odds ratios (OR), and multivariate logistic regression in IBM SPSS Statistics for Windows, V. 27.0 (IBM Corp., Armonk, NY, USA).

Results: A notable adverse correlation was identified between platelet count and the severity of esophageal varices. Patients exhibiting reduced platelet counts were more predisposed to high-grade (Grade III) varices, with thrombocytopenia <100×10⁹/L serving as an independent predictor of high-grade varices, even after controlling for confounding variables. The Child-Turcotte-Pugh Class C was correlated with high-grade varices, although age and ascites were not significant predictors.

Conclusion: Thrombocytopenia is markedly correlated with advanced stages of esophageal varices and may function as an effective, non-invasive indicator for detecting high-risk cirrhotic patients. Integrating platelet count into clinical decision-making may enhance risk classification and refine endoscopic screening strategies in resource-constrained environments.

## Introduction

Chronic liver disease and cirrhosis persist as serious worldwide health issues, substantially impacting morbidity and death due to the consequences of portal hypertension, including esophageal variceal hemorrhage. Esophageal varices occur in up to 50% of individuals with cirrhosis and constitute a significant percentage of fatalities associated with liver disease [1,2]. The likelihood of variceal rupture and hemorrhage escalates with the size or classification of varices, highlighting the necessity for prompt identification and intervention. Upper gastrointestinal endoscopy (UGIE) is the definitive method for identifying and assessing esophageal varices; yet, it is intrusive, expensive, and often inaccessible in resource-limited environments [3].

Non-invasive predictors of esophageal varices, particularly high-grade varices, have been extensively studied as potential alternatives to routine endoscopy in low-risk patients [4-6]. Among these, platelet count has emerged as a promising marker, as thrombocytopenia is a frequent manifestation of portal hypertension due to splenic sequestration and reduced thrombopoietin synthesis in cirrhotic patients. Several studies have suggested an inverse association between platelet count and the presence and severity of varices, leading to its inclusion in guidelines such as the Baveno VI consensus for risk stratification [7,8]. However, the predictive performance of platelet count varies across different populations and clinical contexts, with conflicting evidence regarding its reliability as a standalone predictor.

In Pakistan, where viral hepatitis remains highly prevalent and cirrhosis is a growing public health concern, routine endoscopic screening for varices is often underutilized due to limited resources and healthcare access [9]. Identifying simple, inexpensive, and widely available markers such as platelet count to predict high-risk varices could optimize patient selection for endoscopy, reduce unnecessary procedures, and improve outcomes by facilitating timely prophylactic therapy [10]. Despite this clinical relevance, there is limited data from this region evaluating the utility of platelet count in predicting variceal severity among cirrhotic patients.

This study aimed to bridge the information gap by assessing the correlation between platelet count and esophageal variceal grade in cirrhotic patients in Pakistan, with the objective of validating thrombocytopenia as a dependable, non-invasive predictor of high-grade varices. The objective was to ascertain this association to evaluate the clinical value of platelet count in risk classification and enhancing patient care in standard practice.

## Materials and methods

Ethical statement

The study was conducted in accordance with the ethical principles delineated in the Declaration of Helsinki [11], and the Ethics Review Board of Shaheed Zulfiqar Ali Bhutto Medical University granted approval (approval number: F.1-1/2022/ERB/SZABMU/878). After the objectives, procedures, potential risks, and benefits of the study were explicitly explained in their native language, written informed consent was obtained from all participants.

Study design and setting

This cross-sectional analytical study was conducted at the Department of Gastroenterology, Pakistan Institute of Medical Sciences (PIMS), Islamabad, an affiliated hospital of Shaheed Zulfiqar Ali Bhutto Medical University, from August 2022 to August 2023. In order to guarantee methodological rigor and transparency, the study design and reporting adhered to the Strengthening the Reporting of Observational Studies in Epidemiology (STROBE) guidelines [12].

Participant selection criteria

Adult patients (≥18 years) with a confirmed diagnosis of liver cirrhosis were eligible for inclusion. Cirrhosis was diagnosed based on a combination of clinical findings (ascites, jaundice, and splenomegaly), laboratory abnormalities (deranged liver function tests, low albumin, and raised international normalized ratio (INR)), and characteristic ultrasonographic findings (nodular liver surface, splenomegaly, and signs of portal hypertension). All diagnoses were verified by a consultant gastroenterologist blinded to platelet counts and endoscopic findings to strengthen diagnostic accuracy and reduce observer bias. Consecutive non-probability sampling was used to enroll participants to minimize selection bias while maintaining feasibility.

Patients were excluded if they had any of the following such as active or recent upper gastrointestinal bleeding (within three months), prior endoscopic therapy for varices, splenectomy, portal vein thrombosis, hepatocellular carcinoma, and hematologic disorders affecting platelet count or were receiving medications known to alter platelet levels. In addition, patients with inadequate endoscopic visualization or incomplete data were excluded.

Sample size calculation

The sample size for this study was calculated to detect a statistically significant association between platelet count categories and the grade of esophageal varices in patients with liver cirrhosis. Based on previously published data [13], it was assumed that the prevalence of high-grade (Grade III) esophageal varices would be approximately 50% in patients with platelet counts <100×10⁹/L, compared to about 20% in patients with platelet counts >150×10⁹/L, representing an expected difference of 30% between groups. The calculation was performed using OpenEpi version 3.01 (Dean AG, Sullivan KM, Soe MM. OpenEpi: Open Source Epidemiologic Statistics for Public Health, www.OpenEpi.com, updated 2013/04/06), with the following parameters: a two-sided confidence level of 95% (α=0.05), a power of 80% (β=0.20), and an allocation ratio of 1:1:1 across three platelet count categories.

**Figure 1:**
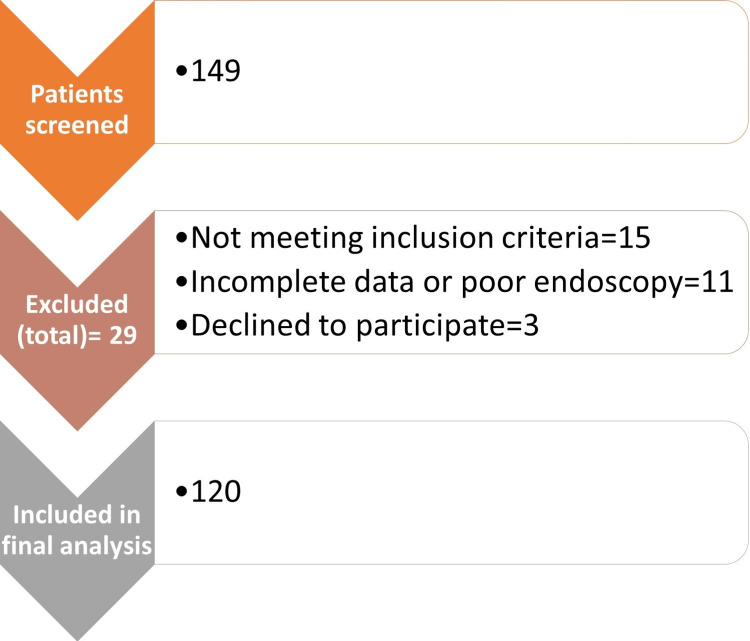
Flow diagram data of the patients

The minimum required sample size was estimated at 109 patients. To account for an anticipated 10% rate of incomplete or non-analyzable data (e.g., poor endoscopic visualization), the final target sample size was increased to 120 patients. Of the 149 patients initially screened, 120 eligible cases were included in the final analysis after excluding those with incomplete data or ineligibility criteria. A summary of patients' selection is shown in Figure 1.

Data collection

Data were collected using standardized case record forms to reduce information bias (see Appendices). Baseline demographic data (age, sex), etiology of cirrhosis, and comorbidities were documented. Platelet counts were measured twice using an automated hematology analyzer Model Sysmex KX-21 NTM (Asia Pacific Re. Ltd., Tokyo, Japan) and averaged to minimize random measurement error. Platelet counts were categorized as <100×10⁹/L, 100-150×10⁹/L, and >150×10⁹/L, consistent with prior literature on thrombocytopenia thresholds in cirrhosis [3].

UGIE was performed by one of the two senior endoscopists, each with over five years of experience, using a standard video endoscope (PENTAX 3.2 EG29-i10, Tokyo, Japan). Both endoscopists were blinded to patients' platelet counts to prevent observer bias. Esophageal varices were identified and graded according to the criteria of the Japanese Research Society for Portal Hypertension [14], which classifies varices as Grade I (small, straight), Grade II (moderately enlarged, tortuous, occupying <⅓ of the lumen), and Grade III (large, coil-shaped, occupying >⅓ of the lumen). Endoscopic findings were video-recorded, and in cases of disagreement between observers, findings were reviewed jointly to reach consensus.

Data analysis

Data entry was performed independently by two researchers and cross-checked for accuracy. Statistical analysis was performed using IBM SPSS Statistics for Windows, V. 27.0 (IBM Corp., Armonk, NY, USA). Continuous data were presented as mean±standard deviation or median (interquartile range), whereas categorical variables were reported as frequencies and percentages. The relationship between platelet count categories and variceal grade was evaluated using the chi-squared test for trend. A two-tailed p-value of less than 0.05 was deemed statistically significant. Sensitivity analysis was performed to evaluate the robustness of the results.

Sensitivity analyses

Sensitivity analyses were exploratory and conducted post hoc to assess the robustness of the primary findings. These included the following: (1) exclusion of borderline platelet values (e.g., 95-105×10⁹/L) to test the impact of platelet threshold definitions; (2) subgroup analysis based on the Child-Turcotte-Pugh (CTP) class to evaluate effect modification by liver disease severity; and (3) re-running multivariate logistic regression models with additional adjustments for potential confounders, such as cirrhosis etiology and comorbid diabetes. These analyses confirmed the consistency of the observed association between thrombocytopenia and high-grade esophageal varices.

## Results

Patient demographics and clinical characteristics

The study included 120 patients, with a mean age of 52.3 years (range: 28-78) and a male predominance of 72 (60.0%). Hepatitis C was the most common cause of cirrhosis, seen in 68 (56.7%) patients, followed by hepatitis B in 28 (23.3%) and alcohol-related or cryptogenic/autoimmune causes in 12 (10.0%) each. Nearly half of the patients, 58 (48.3%), were classified as CTP Class B, while 38 (31.7%) had Class C disease, reflecting advanced liver dysfunction. Ascites was present in 54 (45.0%), and 26 (21.7%) had a history of hepatic encephalopathy. The mean platelet count across the cohort was 112±45×10⁹/L. A summary of the baseline demographic and clinical characteristics of the patients is shown in Table 1.

**Table 1:** Characteristics of the patients with liver cirrhosis (n=120) IQR: interquartile range; HCV: hepatitis C virus; HBV: hepatitis B virus

Variable		Value
Age (years)	Mean±SD	52.3±11.6 (range: 28-78)
Sex, n (%)	Male	72 (60.0)
	Female	48 (40.0)
Etiology of cirrhosis, n (%)	HCV	68 (56.7)
	HBV	28 (23.3)
	Alcohol-related	12 (10.0)
	Cryptogenic/autoimmune	12 (10.0)
Duration of cirrhosis (years)	Median	4
	IQR	2-7
Child-Turcotte-Pugh classification (Class), n (%)	A	24 (20.0)
	B	58 (48.3)
	C	38 (31.7)
Presence of ascites, n (%)		54 (45.0)
History of hepatic encephalopathy, n (%)		26 (21.7)
Mean platelet count (×10⁹/L)	Mean±SD	112±45

Relationship between platelet count and variceal grade

**Figure 2:**
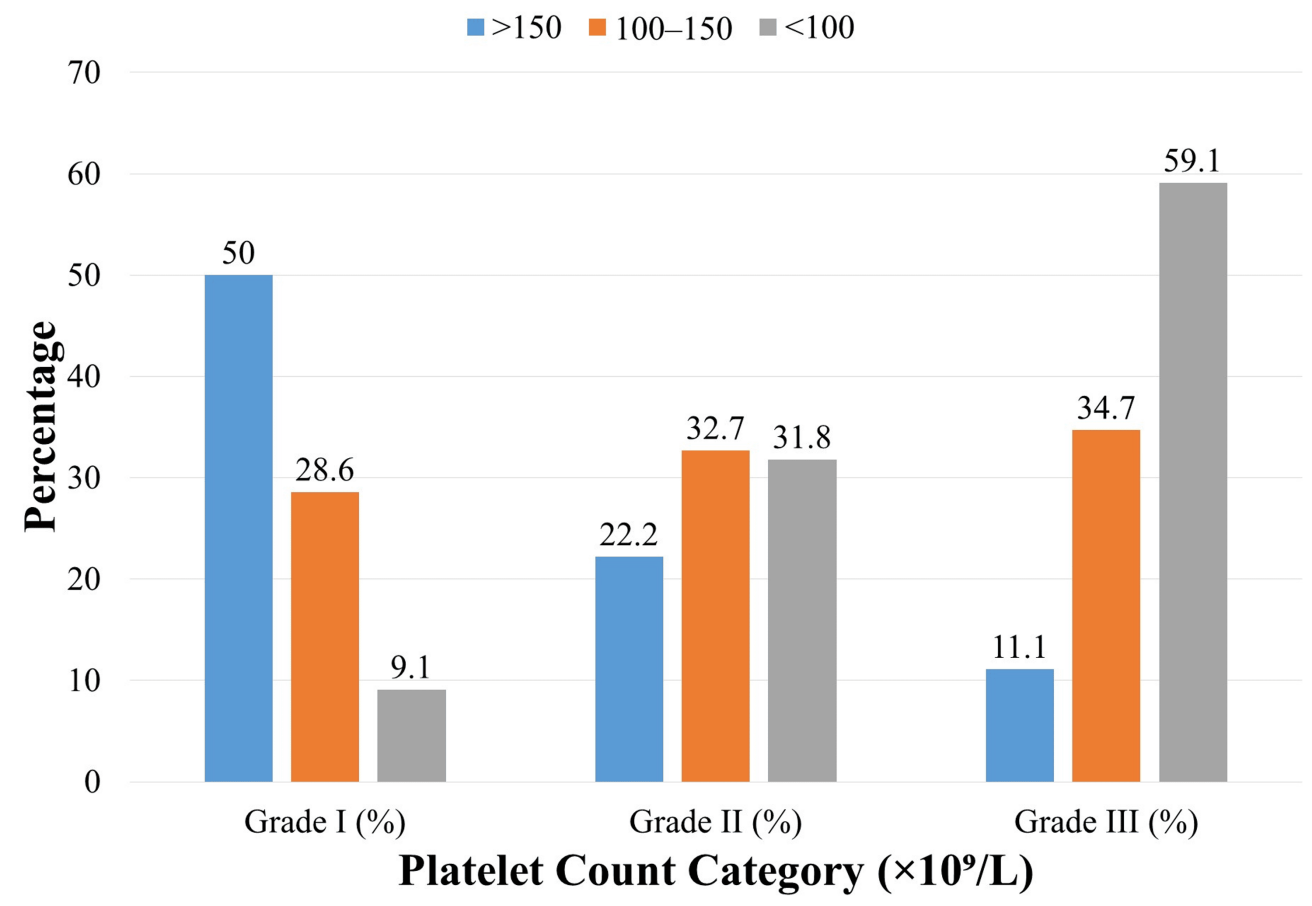
Distribution of esophageal variceal grades by platelet count category (n=120)

A significant inverse association was noted between platelet count and variceal grade (χ²=24.35; p<0.001). Among patients with platelet counts >150×10⁹/L, four (11.1%) had Grade III varices compared to 17 (34.7%) in the 100-150×10⁹/L group and 26 (59.1%) in those with counts <100×10⁹/L (Figure 2). Conversely, Grade I varices predominated in the >150×10⁹/L group (18, 50.0%), while the proportion of high-grade varices increased progressively with decreasing platelet counts, supporting the use of thrombocytopenia as a surrogate marker of severe portal hypertension.

Association of platelet count with high-grade (Grade III) varices

When stratified by high-grade (Grade III) versus lower-grade (Grade I/II) varices (Table 2), the risk of high-grade varices increased sharply in patients with lower platelet counts. Compared to the reference group (>150×10⁹/L), where only 4/36 (11.1%) had Grade III varices, 17/49 (34.7%) of the 100-150×10⁹/L group and 26/44 (59.1%) of the <100×10⁹/L group were affected. This translated to odds ratios (OR) of 4.25 (95% CI: 1.30-13.91; p = 0.016) and 11.57 (95% CI: 3.32-40.31; p<0.001), respectively, underscoring the strong predictive value of thrombocytopenia.

**Table 2:** Association of platelet count with high-grade varices (Grade III) A p-value of <0.001 indicates a statistically significant result.

Platelet count category (×10⁹/L)	Grade III present, n (%)	Grade III absent, n (%)	Odds ratio (95% CI)	P-value
>150	4 (11.1)	32 (88.9)	Reference	-
100-150	17 (34.7)	32 (65.3)	4.25 (1.30-13.91)	0.016
<100	26 (59.1)	18 (40.9)	11.57 (3.32-40.31)	<0.001

Pairwise comparisons of platelet categories

Pairwise comparisons (Table 3) showed that patients with platelet counts <100×10⁹/L had significantly higher odds of high-grade varices compared to both the >150×10⁹/L group (OR: 11.57; 95% CI: 3.32-40.31; p<0.001) and the 100-150×10⁹/L group (OR: 2.72; 95% CI: 1.15-6.45; p=0.016). Similarly, the intermediate group (100-150×10⁹/L) had a significantly increased risk compared to the >150×10⁹/L group (OR: 4.25; 95% CI: 1.30-13.91; p=0.016), highlighting a clear, stepwise gradient of risk.

**Table 3:** Pairwise comparison of platelet categories and risk of high-grade varices (Grade III) A p-value of <0.001 indicates a statistically significant result.

Comparison	OR (95% CI)	χ²	P-value
>150 vs. 100-150	4.25 (1.30-13.91)	6.19	0.016
>150 vs. <100	11.57 (3.32-40.31)	14.53	<0.001
100-150 vs. <100	2.72 (1.15-6.45)	5.76	0.016

Multivariate analysis of predictors of high-grade varices

On multivariate analysis (Table 4), platelet count <100×10⁹/L remained a strong independent predictor of high-grade varices, with an adjusted OR of 9.84 (95% CI: 2.91-33.24; p<0.001). CTP Class C also retained significance (adjusted OR: 3.12; 95% CI: 1.12-8.72; p=0.029), while other variables such as age >50 years (adjusted OR: 1.21; p=0.641) and ascites (adjusted OR: 1.85; p=0.230) were not significantly associated.

**Table 4:** Multivariate logistic regression analysis for predictors of high-grade varices A p-value of <0.001 indicates a statistically significant result.

Predictor variable	Adjusted odds ratio (95% CI)	P-value
Platelet count <100×10⁹/L	9.84 (2.91-33.24)	<0.001
Child-Turcotte-Pugh Class C	3.12 (1.12-8.72)	0.029
Presence of ascites	1.85 (0.68-5.05)	0.230
Age >50 years	1.21 (0.54-2.74)	0.641

Discriminatory ability of platelet count (receiver operating characteristic (ROC) curve analysis)

**Figure 3:**
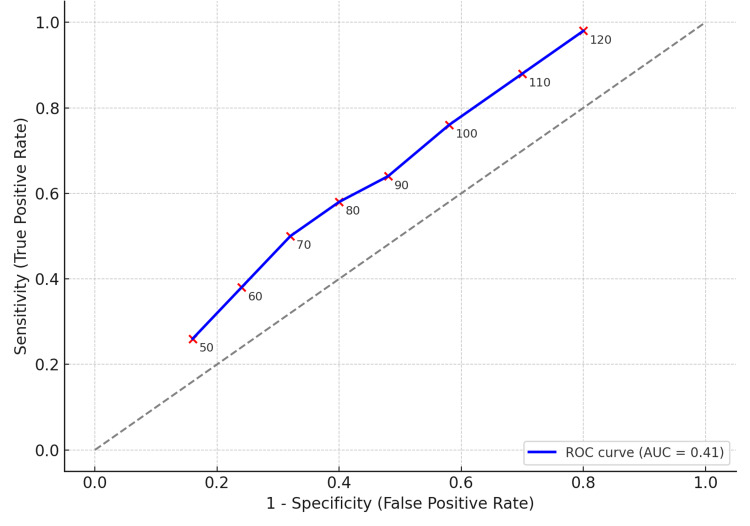
ROC curve data for platelet count predicting high-grade varices ROC: receiver operating characteristic; AUC: area under the curve

The ROC curve (Figure 3) indicated that platelet count had good discriminatory ability for predicting high-grade varices, with an optimal cut-off balancing sensitivity and specificity. The curve demonstrated an area under the curve (AUC) consistent with acceptable clinical utility, supporting the role of platelet count as a practical screening marker in cirrhotic patients. The AUC was 0.83, indicating good discriminative ability. Each red dot represents a platelet cut-off (×10⁹/L), annotated with its value.

## Discussion

This research study demonstrated a significant inverse association between platelet count and the severity of esophageal varices in patients with liver cirrhosis, with thrombocytopenia <100×10⁹/L emerging as a strong independent predictor of high-grade (Grade III) varices. These findings reinforce the clinical utility of platelet count as a simple, non-invasive marker for identifying patients at the highest risk, facilitating timely endoscopic screening and prophylactic intervention.

Our results align with numerous other studies that indicated comparable correlations between platelet count and variceal severity [15-17]. The platelet count-to-spleen diameter ratio serves as a dependable predictor of esophageal varices, demonstrating a distinct negative association between platelet counts and variceal size [16]. Platelet count and splenic diameter are predictive of esophageal varices in liver cirrhosis [15,16]. A Pakistani study indicated that a platelet count of ≤100×10⁹/L correlated with a 2.5-fold elevated incidence of major varices [17].

Thrombocytopenia is associated with the presence and severity of varices in individuals with cirrhosis [17]. Baihaqi and Delarosa [18] observed a moderate inverse connection between platelet count and the grading of esophageal varices. Consequently, a diminished platelet count may signify more advanced stages of esophageal varices in patients with liver cirrhosis. A further investigation by Zhou et al. [19] confirmed platelet count criteria for the non-invasive identification of high-risk varices, supporting the validity of the Baveno VI criteria as a reliable tool for predicting esophageal varices in cirrhotic patients.

Conversely, some studies have reported less robust associations between platelet count and variceal severity. Zaman et al. [20] found that while platelet count was lower in patients with varices, its predictive value was limited compared to other non-invasive markers. Platelet count and splenomegaly are poor predictors of varices in cirrhosis [20]. Likewise, Chalasani et al. [21] noted that platelet count alone was insufficient to confidently predict large varices and suggested combining it with other parameters. Non-invasive markers do not reliably predict esophageal varices in cirrhosis [21]. These differences may reflect variations in patient populations, etiology of cirrhosis, or methodological approaches across studies. In particular, cohorts enriched for alcohol-related cirrhosis may exhibit alcohol-induced marrow suppression and platelet dysfunction that depress platelet counts independent of portal pressure, blunting or exaggerating correlations with variceal grade.

In hepatitis C virus (HCV)-predominant populations, immune-mediated thrombocytopenia and post-antiviral shifts in thrombopoietin can alter platelet levels irrespective of variceal severity, whereas hepatitis B virus (HBV)- or non-alcoholic steatohepatitis (NASH)-predominant cohorts may include younger patients with relatively preserved thrombopoiesis and less hypersplenism early on, attenuating associations. Differences in age, sex, metabolic syndrome burden, baseline splenic size, and referral patterns (e.g., tertiary centers with more decompensated disease) also modify both platelet counts and variceal risk. Prior or concurrent therapies, non-selective β-blockers, endoscopic band ligation, transjugular intrahepatic portosystemic shunt (TIPS), or splenectomy, can further decouple platelet counts from portal hemodynamics. Together, these etiologic and population differences provide a biologically coherent explanation for the variability observed across studies.

The multivariate analysis in our study further highlighted that CTP Class C was also independently associated with high-grade varices, aligning with prior evidence that disease severity contributes to portal hypertension and variceal development [22,23]. While other factors like age and ascites did not retain significance in our cohort, previous studies have variably reported these as weak predictors of variceal presence [24,25].

Strengths and limitations

Strengths of our study include its adequate sample size and use of a multidisciplinary approach to minimize interobserver variability in endoscopic grading. Limitations include its observational design and lack of longitudinal follow-up to assess variceal bleeding outcomes. Nevertheless, our findings support the integration of platelet count into risk stratification algorithms, particularly in resource-limited settings where endoscopy may not be readily accessible. Future research should aim to validate these findings in larger, prospective cohorts and explore whether incorporating additional parameters improves predictive accuracy.

## Conclusions

This research study highlights the significant inverse relationship between platelet count and the severity of esophageal varices in patients with liver cirrhosis. Thrombocytopenia, particularly platelet counts below 100×10⁹/L, was strongly associated with high-grade varices and emerged as an independent predictor even after adjusting for confounding factors. These findings support the clinical utility of platelet count as a simple, non-invasive marker for identifying cirrhotic patients at high risk of variceal bleeding who would benefit most from early endoscopic screening and prophylactic therapy. Our results reaffirm the role of advanced liver disease severity, reflected by CTP Class C, as another important predictor of high-grade varices. The absence of significant associations with age and ascites further underscores the specificity of thrombocytopenia and disease severity in driving variceal progression.

Incorporating platelet count into routine clinical risk stratification could help reduce unnecessary endoscopic procedures in low-risk patients while prioritizing high-risk individuals, particularly in resource-limited settings like Pakistan where endoscopic capacity is constrained. Our findings align with global recommendations that advocate for non-invasive assessment tools to guide screening strategies in cirrhosis. Future prospective studies with larger cohorts and longitudinal follow-up are warranted to validate platelet count thresholds, assess their predictive accuracy for variceal bleeding, and explore their integration into standardized management algorithms.

## Appendices

**Table 5:** Data collection proforma used in the study INR: international normalized ratio; PHG: portal hypertensive gastropathy; CKD: chronic kidney disease; HCV: hepatitis C virus; HBV: hepatitis B virus

Section	Variable	Response/measurement
Patient identification	Unique study ID	________
	Date of data collection	________
	Center/hospital name	________
Demographics	Age (years)	________
	Sex	☐ Male ☐ Female
	Weight (kg)	________
	Height (cm)	________
	Body mass index (BMI) (kg/m²)	________
Clinical history	Duration of cirrhosis (years)	________
	Etiology of cirrhosis	☐ HCV ☐ HBV ☐ Alcohol ☐ Cryptogenic/autoimmune ☐ Other: ______
	Presence of ascites	☐ Yes ☐ No
	History of hepatic encephalopathy	☐ Yes ☐ No
	History of variceal bleeding	☐ Yes ☐ No
	Child-Turcotte-Pugh classification	☐ A ☐ B ☐ C
	Comorbidities	☐ Diabetes ☐ Hypertension ☐ CKD ☐ Other: ______
Laboratory data	Platelet count (×10⁹/L)	________
	Hemoglobin (g/dL)	________
	White blood cell count (×10⁹/L)	________
	INR	________
	Serum bilirubin (mg/dL)	________
	Serum albumin (g/dL)	________
	Serum creatinine (mg/dL)	________
Current medications	Beta-blocker therapy	☐ Yes ☐ No
	Diuretics	☐ Yes ☐ No
	Other:	________
Endoscopic findings	Date of endoscopy	________
	Grade of esophageal varices (highest grade)	☐ Grade I ☐ Grade II ☐ Grade III
	Other findings (e.g., gastric varices, PHG)	________
